# Bis[*N*-(2-hy­droxy­eth­yl)-*N*-iso­propyl­dithio­carbamato-κ^2^ *S*,*S*′](piperazine-κ*N*)cadmium: crystal structure and Hirshfeld surface analysis

**DOI:** 10.1107/S2056989016000165

**Published:** 2016-01-13

**Authors:** Siti Artikah M. Safbri, Siti Nadiah Abdul Halim, Mukesh M. Jotani, Edward R. T. Tiekink

**Affiliations:** a Department of Chemistry, University of Malaya, 50603 Kuala Lumpur, Malaysia; b Department of Physics, Bhavan’s Sheth R. A. College of Science, Ahmedabad, Gujarat 380001, India; c Centre for Crystalline Materials, Faculty of Science and Technology, Sunway University, 47500 Bandar Sunway, Selangor Darul Ehsan, Malaysia

**Keywords:** crystal structure, Hirshfeld surface analysis, di­thio­carbamate, piperazine, hydrogen bonding

## Abstract

The title compound, [Cd(C_6_H_12_NOS_2_)_2_(C_4_H_10_N_2_)], features a distorted square-pyramidal coordination geometry about the central Cd^II^ atom. The di­thio­carbamate ligands are chelating, forming similar Cd—S bond lengths and define the approximate basal plane. One of the N atoms of the piperazine mol­ecule, which adopts a chair conformation, occupies the apical site. In the crystal, supra­molecular layers propagating in the *ac* plane are formed *via* hy­droxy-O—H⋯O(hy­droxy), hy­droxy-O—H⋯N(terminal-piperazine) and coordinated-piperazine-N—H⋯O(hy­droxy) hydrogen bonds; the layers also feature methine-C—H⋯S inter­actions and S⋯S [3.3714 (10) Å] short contacts. The layers stack along the *b-*axis direction with very weak terminal-piperazine-N—H⋯O(hy­droxy) inter­actions between them. An evaluation of the Hirshfeld surfaces confirms the importance of inter­molecular inter­actions involving oxygen and sulfur atoms.

## Abstract 2

A distorted square-pyramidal CdNS_4_ coordination geometry is found in {Cd[S_2_CN(^*i*^Pr)CH_2_CH_2_OH]_2_[HN(CH_2_CH_2_)_2_NH]}. The packing features supra­molecular layers sustained by O—H⋯O, O—H⋯N and N—H⋯O hydrogen bonding.

## Chemical context   

In the solid state, binary bis­(di­thio­carbamato) compounds of cadmium are usually binuclear with five-coordinate geometries owing to the presence of equal numbers of chelating and μ_2_–tridentate ligands, *i.e*. are of general formula [Cd(S_2_CN*R*_2_)_2_]_2_ (Tiekink, 2003 ▸). Equally well known is the observation that upon the addition of base, this motif is disrupted, resulting in mononuclear species, such as in the case of the pyridine adduct, {Cd[S_2_CN(CH_2_C(H)Me_2_)_2_]_2_(pyridine)} (Rodina *et al.*, 2011 ▸). Ditopic donors can give rise to zero- or one-dimensional species. Thus, when the bidentate ligand is capable of chelating, mononuclear species are obtained, *e.g*. [Cd(S_2_CN(Me)^*i*^Pr)_2_(2,2′-bi­pyridine)] (Wahab *et al.*, 2011 ▸). More variety is found in adducts containing mol­ecules capable of bridging where zero-dimensional binuclear structures, *e.g*. [Cd(S_2_CNPr_2_)_2_(2-pyridine­aldazine)]_2_ (Poplaukhin & Tiekink, 2008 ▸), or supra­molecular chains, *e.g*. [Cd(S_2_CNEt_2_)_2_(μ_2_-1,2-bis­(4-pyrid­yl)ethyl­ene)]_*n*_ (Chai *et al.*, 2003 ▸), are found. An intriguing structure has been reported where the potentially μ_2_-bridging ligand, 4-pyridine­aldazine, coordinates in the monodentate mode in {Cd[S_2_CN(Pr)CH_2_CH_2_OH]_2_(4-pyridine­aldazine)_2_} (Broker & Tiekink, 2011 ▸). The latter, featuring a di­thio­carbamate ligand functionalized with a hy­droxy­ethyl substituent capable of hydrogen bonding, has sparked systematic studies of their wider structural chemistry, revealing hitherto unobserved structural motifs for cadmium di­thio­carbamates (Tan *et al.*, 2013 ▸, 2016 ▸). As a continuation of this work, the title compound was investigated where both the di­thio­carbamate ligand and the nitro­gen-donor ligand have hydrogen-bonding potential.
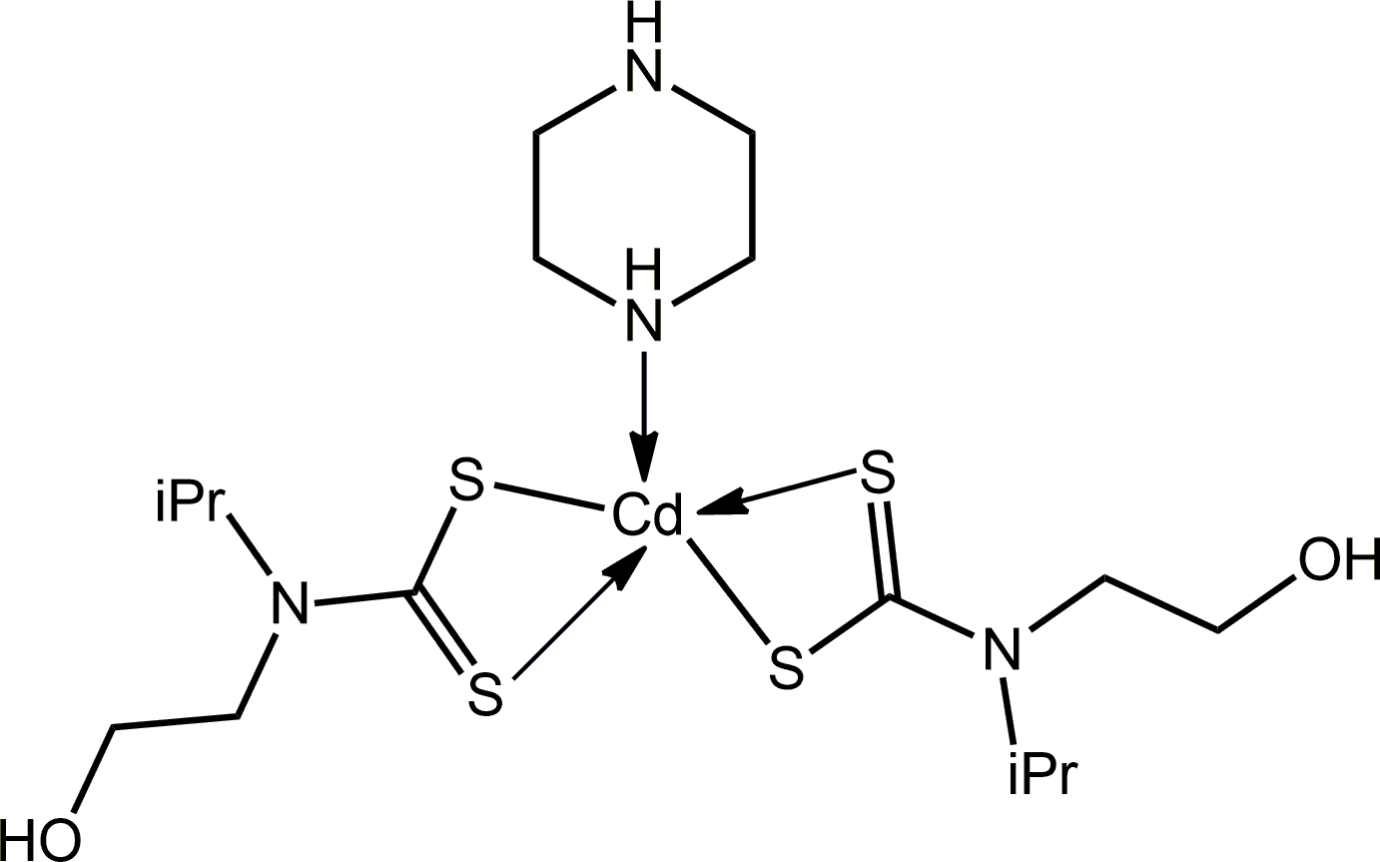


## Structural commentary   

**Figure 1:**
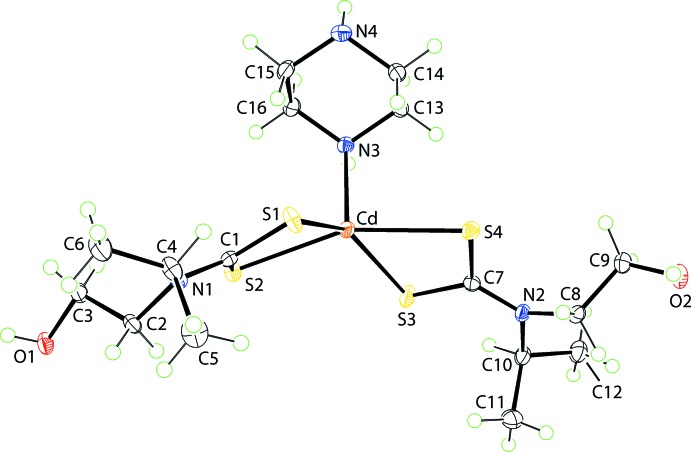
The mol­ecular structure of the title compound, {Cd[S_2_CN(^*i*^Pr)CH_2_CH_2_OH]_2_[HN(CH_2_CH_2_)_2_NH]}, showing the atom-labelling scheme and displacement ellipsoids at the 50% probability level.

The mol­ecular structure of the title compound, {Cd[S_2_CN(^*i*^Pr)CH_2_CH_2_OH]_2_[HN(CH_2_CH_2_)_2_NH]}, Fig. 1 ▸, comprises a penta-coordinated cadmium atom, being chelated by two di­thio­carbamate ligands and connected to a piperazine-N atom, the latter ligand having a chair conformation. The coordination geometry is best described as being distorted square pyramidal with the nitro­gen atom in the apical position. This description is qu­anti­fied by the value of τ, *i.e*. 0.18, which is closer to the τ value of 0.0 for an ideal square-pyramidal geometry *cf*. 1.0 for an ideal trigonal bipyramid (Addison *et al.*, 1984 ▸). The r.m.s. deviation of the four sulfur atoms is 0.1023 Å, and the cadmium atom lies 0.6570 (4) Å above the plane in the direction of the N3 atom. The distortions from the ideal geometry are related, in part, to the acute chelate angles subtended by the chelating ligands, *i.e*. 68–69°, and the range of N_axial_—Cd—S_basal_ angles is 98–116°, Table 1 ▸. The di­thio­carbamate ligands are coordinating in a slightly asymmetric manner with the difference between the short and long Cd—S bond lengths being *ca* 0.1 Å for the S1-containing ligand and *ca* 0.2 Å for the S3-containing ligand, Table 1 ▸. The almost symmetric mode of coordination of the di­thio­carbamate ligands is reflected in the near equivalence of the associated C—S bond lengths, Table 1 ▸, and is consistent with significant delocalization of π-electron density over each four-membered chelate ring.

The monodentate mode of coordination of the piperazine ligand in the title compound is without precedent in the crystallographic literature of cadmium (Groom & Allen, 2014 ▸). However, there are several examples of bridging piperazine, *e.g*. [CdBr_2_(μ_2_-piperazine)]_*n*_ (Yu *et al.*, 2007 ▸), {Cd[1,3-(CO_2_)_2_C_6_H_4_](μ_2_-piperazine)(OH_2_)}_*n*_ (Gu *et al.*, 2011 ▸) and [Cd(SCN)_2_(μ_2_-piperazine)]_*n*_ (Suen & Wang, 2007 ▸). In common with the title compound, the piperazine ring adopts a chair conformation in each of these structures.

## Supra­molecular features   

**Figure 2:**
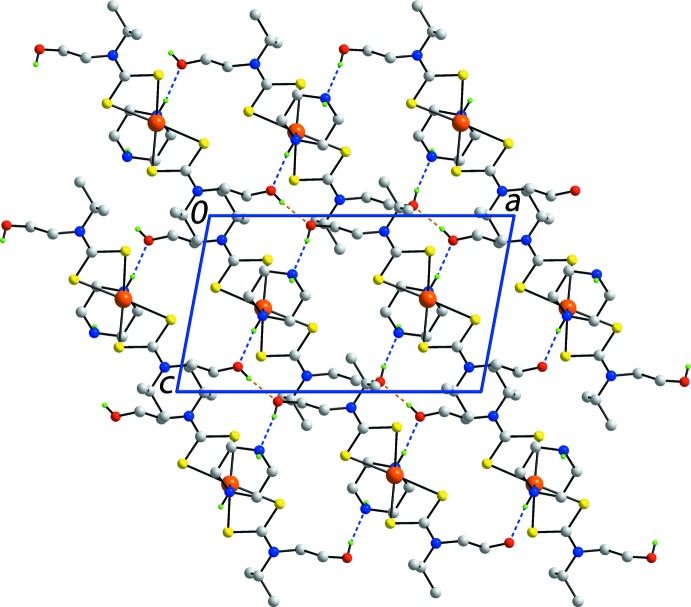
A view of the supra­molecular layer in the title compound, shown in projection down the *b* axis. The hy­droxy-O1—H⋯O2(hy­droxy) hydrogen bonds are shown as orange dashed lines while both the hy­droxy-O2—H⋯N4(terminal-piperazine) and (coordinated-piperazine)-N3—H⋯O1(hy­droxy) hydrogen bonds are shown as blue dashed lines. The methine-C—H⋯S and S⋯S inter­actions within the layers (see text) are not shown. Only acidic hydrogen atoms are shown.

**Figure 3:**
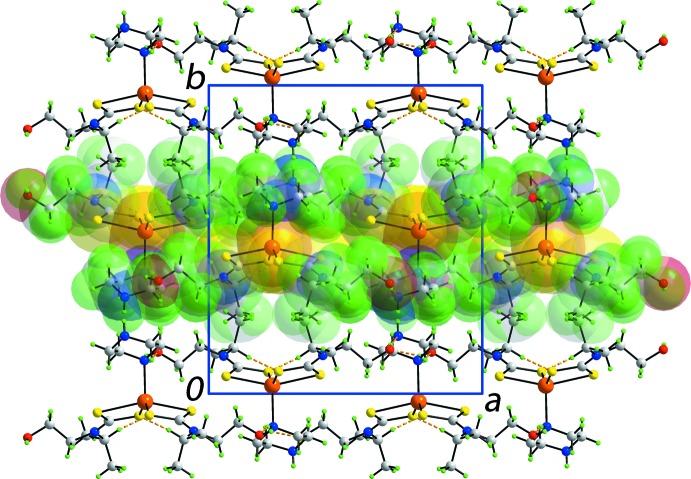
A view of the unit-cell contents of the title compound shown in projection down the *c* axis, whereby the supra­molecular layers, illustrated in Fig. 2 ▸, stack along the *b* axis. One layer is highlighted in space-filling mode.

In the extended structure, hy­droxy-O1—H⋯O2(hy­droxy), hy­droxy-O2—H⋯N4(terminal-piperazine) and (coordinated-piperazine)-N3—H⋯O1(hy­droxy) hydrogen bonds (Table 2 ▸) lead to the formation of a supra­molecular layer in the *ac* plane, Fig. 2 ▸. Additional stability to the layers are afforded by methine-C—H⋯S1, S3 contacts, Table 2 ▸, as well as S2⋯S2^i^ contacts of 3.3714 (10) Å, for symmetry operation: (i) 2 − *x*, 1 − *y*, 1 − *z*. Layers thus formed stack along the *b* axis, with very weak terminal-piperazine-N4—H⋯O2(hy­droxy) inter­actions between them, Table 2 ▸ and Fig. 3 ▸.

## Analysis of the Hirshfeld surfaces   

The program *Crystal Explorer* (Wolff *et al.*, 2012 ▸) was used to generate Hirshfeld surfaces mapped over *d*_norm_, *d_e_* and electrostatic potential for the title compound. The electrostatic potentials were calculated using *TONTO* (Spackman *et al.*, 2008 ▸; Jayatilaka *et al.*, 2005 ▸) and were mapped on Hirshfeld surfaces using the STO-3G basis set at the Hartree–Fock level of theory over a range ±0.14 au. The contact distances *d_i_* and *d_e_* from the Hirshfeld surface to the nearest atom inside and outside, respectively, enable the analysis of the inter­molecular inter­actions through the mapping of *d*_norm_. The combination of *d_e_* and *d_i_* in the form of a two-dimensional fingerprint plot (Rohl *et al.*, 2008 ▸) provides a summary of inter­molecular contacts in the crystal. The relative contributions from various contacts to the Hirshfeld surfaces are tabulated in Table 3 ▸.

**Figure 4:**
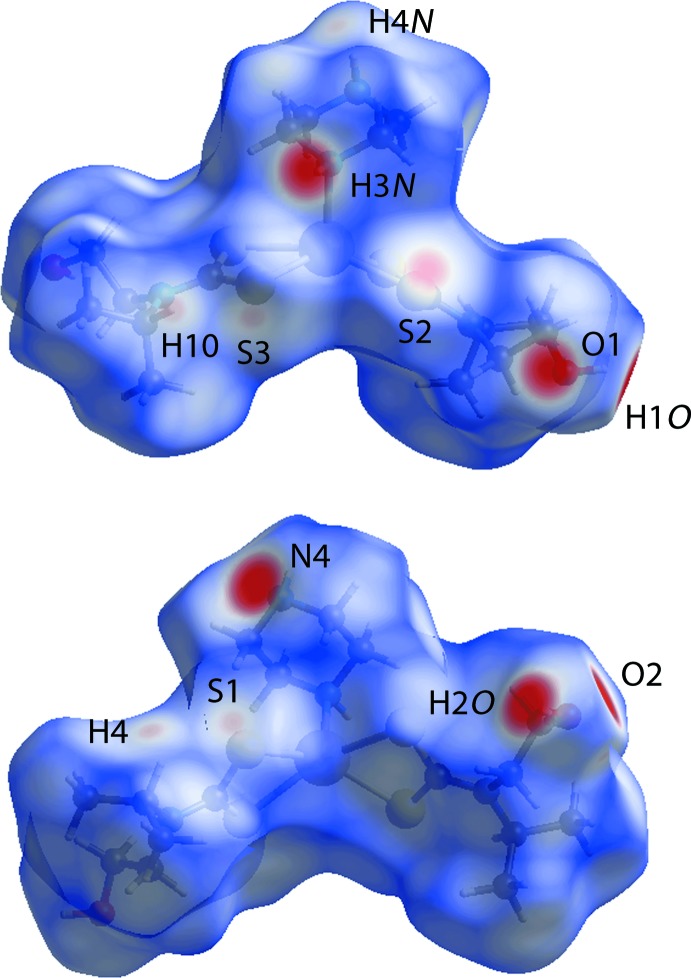
Two views of the Hirshfeld surface mapped over *d*_norm_. The contact points (red) are labelled to indicate the atoms participating in the inter­molecular inter­actions.

**Figure 5:**
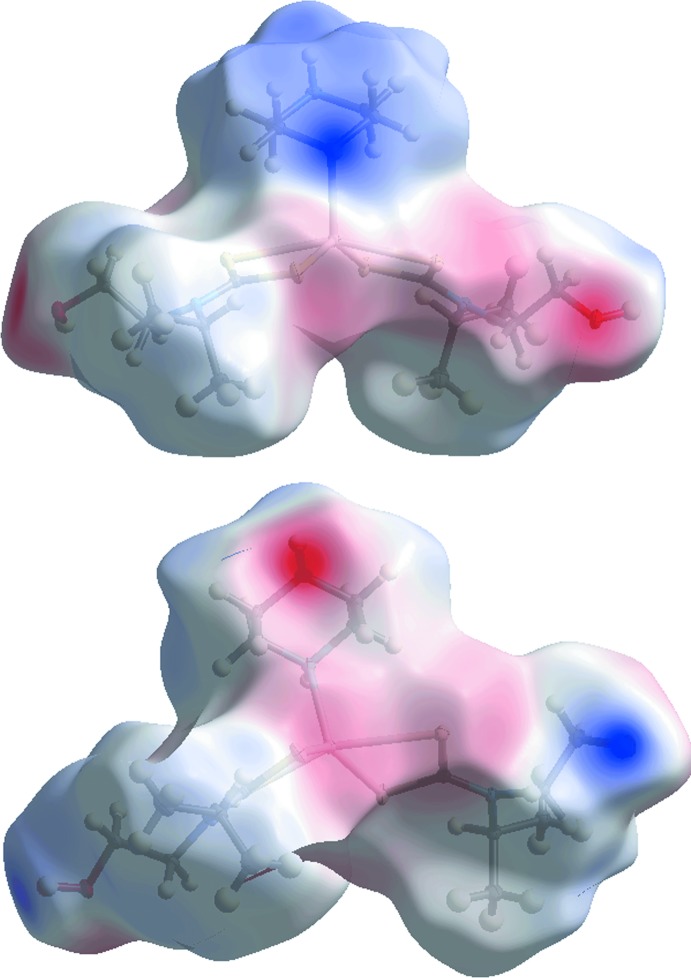
Two views of the Hirshfeld surface mapped over the electrostatic potential with positive and negative potential indicated in blue and red, respectively.

**Figure 6:**
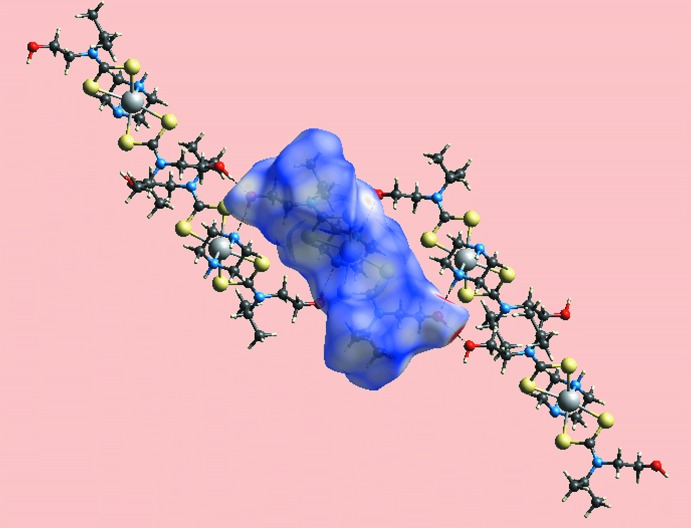
Hirshfeld surface mapped for a reference mol­ecule over *d*_norm_ showing hydrogen bonds with neighbouring mol­ecules.

The hydrogen-bonding network generated in the crystal through hydroxyl groups located at the edges, piperazine nitro­gen-H at the apex and sulfur atoms on the vertices of the distorted square-pyramidal polyhedron can be visualized using Hirshfeld surface analysis. The bright-red spots on the Hirshfeld surface mapped over *d*_norm_, Fig. 4 ▸, with labels H1*O*, H2*O* and H3*N*, on the surface represent donors for potential hydrogen bonds, Table 2 ▸; the corresponding acceptors on the surfaces appear as bright-red spots at O2, N4 and O1, respectively. The Hirshfeld surface mapped over the electrostatic potential, Fig. 5 ▸, represents donors with positive potential (blue regions) and the acceptors with negative potential (red). In addition, the negative potential around the sulfur atoms appear as light-red clouds and the positive potential around piperazine as a light-blue cloud in Fig. 5 ▸. The Hirshfeld surfaces mapped over *d*_norm_ showing inter­molecular O—H⋯O, N—H⋯O and O—H⋯N bonds with symmetry-related mol­ecules are shown in Fig. 6 ▸. The pale-red depressions near the atoms H4 and S1, and H10 and S3, Fig. 4 ▸, confirm the contribution of these pairs of atoms in the comparatively weak inter­molecular C—H⋯S inter­actions. The pale-red spot near the S2 atom indicates an additional reinforcement to the two-dimensional framework through a non-bonded S⋯S contact.

**Figure 7:**
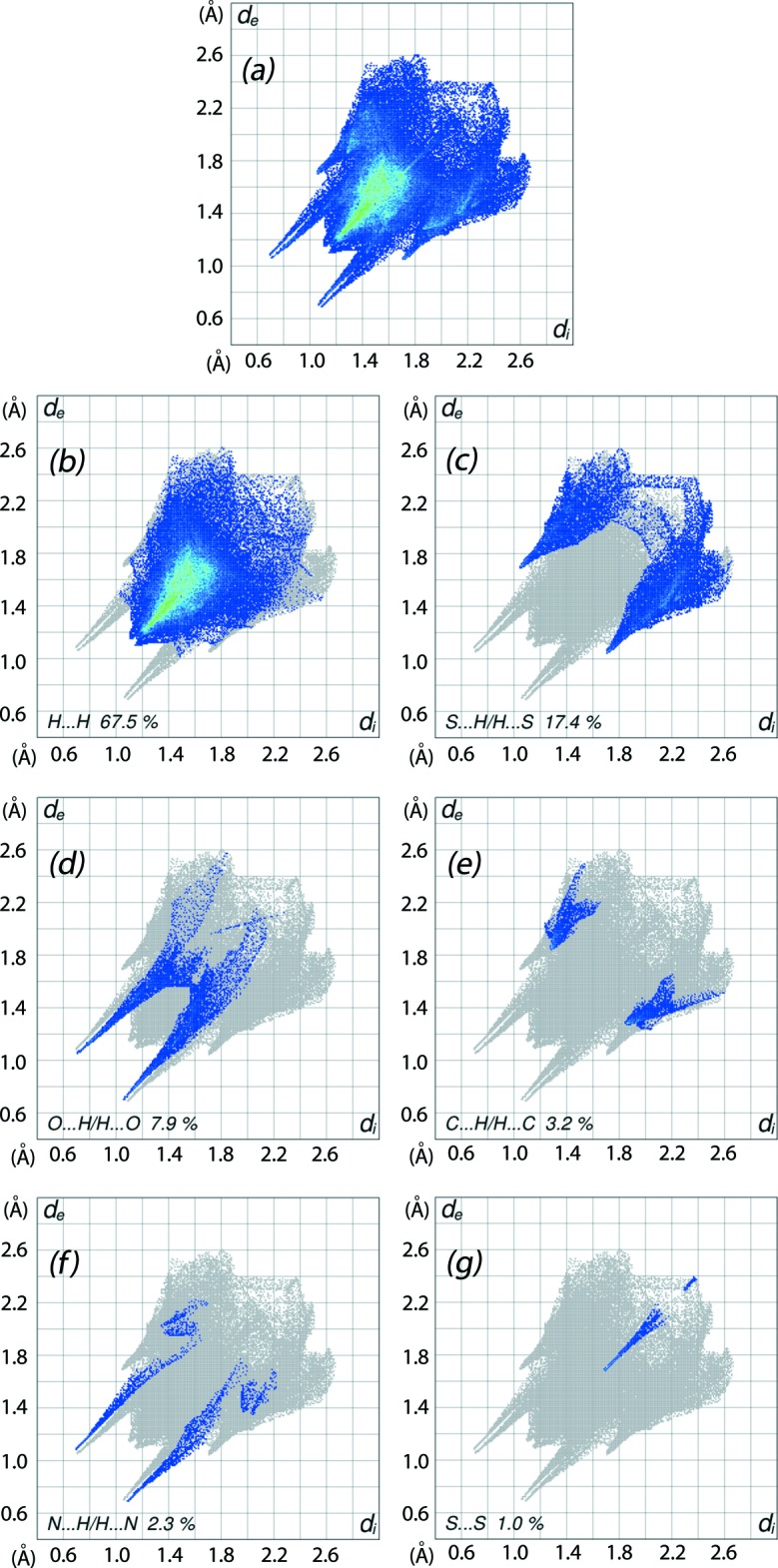
Two-dimensional fingerprint plots: (*a*) overall, and delineated into contributions from different contacts: (*b*) H⋯H, (*c*) S⋯H/H⋯S, (*d*) O⋯H/H⋯O, (*e*) C⋯H/H⋯C, (*f*) N⋯H/H⋯N and (*g*) S⋯S.

The overall two-dimensional fingerprint plot, Fig. 7 ▸ *a*, and those delineated into H⋯H, S⋯H/H⋯S, O⋯H/H⋯O, C⋯H/H⋯C, N⋯H/H⋯N and S⋯S inter­actions are illus­trated in Fig. 7 ▸ *b*–*g*, respectively. The greatest contribution to the overall Hirshfeld surface, *i.e*. 67.5%, is due to H⋯H contacts and is reflected in Fig. 4 ▸ *b* as widely scattered points with a high concentration in the middle region, shown in green. The contribution from the S⋯H/H⋯S contacts, corresponding to C—H⋯S inter­actions, is represented by the pair of short spikes in the outer region, at *d_e_* + *d_i_* ∼ 2.8 Å, Fig. 7 ▸ *c*. In the plots delineated into O⋯H/H⋯O and N⋯H/H⋯N contacts, Fig. 7 ▸ *d* and *f*, the pairs of adjacent peaks have almost same lengths near *d_e_* + *d_i_* ∼ 1.8 Å, and clearly indicate the significance of inter­molecular hydrogen bonds associated with them in the mol­ecular packing. There is only a very small contribution from C⋯H/H⋯C contacts, *i.e*. 3.2% (Fig. 7 ▸ *d*), and there is no contribution from C⋯C contacts in the structure as the result of the absence of C—H⋯π and π–π stacking inter­actions. Finally, the presence of S⋯S contacts can also be viewed in the delineated fingerprint plot, Fig. 7 ▸ *g*, by the density of points in the *d_e_*, *d_i_* region around 1.7–2.4 Å as a broken line segment.

The identified inter­molecular inter­actions were further evaluated by an analysis of the enrichment ratios (ER) that give a qu­anti­tative measure of the likelihood of specific inter­molecular inter­actions to occur based on a Hirshfeld surface analysis (Jelsch *et al.*, 2014 ▸); ratios are given in Table 4 ▸.

A total of 83.2% of the Hirshfeld surface involves hydrogen atoms and of this, non-bonded H⋯H contacts account for 67.5% of the contacts, which is close to the value of 69.2%, being the value calculated for random contacts so that the corresponding ER value is 0.98, *i.e*. near unity and in accord with earlier published results (Jelsch *et al.*, 2014 ▸). The sulfur atoms comprise 9.7% of the surface and S⋯H/H⋯S contacts provide an overall 17.4% contribution to the surface resulting in an ER of 1.1 which is in the expected range, *i.e*. 1.0–1.5, for C—H⋯S inter­actions. The ER value of 1.2 corresponding to O⋯H/H⋯O contacts indicate these show a high propensity to form even though the relative contribution to the overall surface, *i.e*. 7.9%, is small as is the 4.0% exposure to the surface provided by the hydroxyl oxygen atoms. The other contributions to the surface, *i.e*. N⋯H/H⋯N and C⋯H/H⋯C, are small and the ER values are not particularly informative although being > 1, indicate a propensity to form as discussed above in *Supra­molecular features*.

## Database survey   

The structural chemistry of cadmium di­thio­carbamates where the ligands have been functionalized with one or two hy­droxy­ethyl groups has received some attention in recent years owing to the constant stream of unexpected crystallization outcomes. As mentioned in the *Chemical context*, [Cd(S_2_CNR_2_)_2_]_2_ compounds are usually binuclear (Tiekink, 2003 ▸). However, recent studies of Cd[S_2_CN(^*i*^Pr)CH_2_CH_2_OH]_2_ (Tan *et al.*, 2013 ▸, 2016 ▸) have revealed solvent-dependent and solvent-independent supra­molecular mol­ecular isomers, *e.g*. crystallization from ethanol produced two species [Cd[S_2_CN(^*i*^Pr)CH_2_CH_2_OH]_2_·EtOH]_*x*_ for *x* = 2 and, unprecedented, *n* (Tan *et al.*, 2016 ▸), with the kinetic, polymeric (*x* = *n*) form transforming in solution to the thermodynamic, binuclear form (*x* = 2). Other recrystallization conditions led to decomposition of the di­thio­carbamate ligands and subsequent formation of a co-crystal and some salts. This behaviour, along with the unexpected structure of {Cd[S_2_CN(^*i*^Pr)CH_2_CH_2_OH]_2_(4-pyridine­aldazine)_2_] (Broker & Tiekink, 2011 ▸) mentioned in the *Chemical context*, suggests this is a fertile area of crystallographic research. Finally, it is noted that gold (Jamaludin *et al.*, 2013 ▸), zinc (Tan *et al.*, 2015 ▸) and bis­muth (Ishak *et al.*, 2014 ▸) compounds of these ligands display promising potential as anti-cancer agents, and that some gold compounds also exhibit exciting anti-microbial activity (Sim *et al.*, 2014 ▸).

The monodentate mode of coordination of the piperazine ligand in the title compound is quite rare, and has not been observed in the structural chemistry of cadmium. However, crystallographically confirmed examples of a monodentate coordination mode for piperazine have been seen in five structures, *e.g*. as in centrosymmetric, all-*trans* CoCl_2_(piperazine)_2_(MeOH)_2_ (Suen *et al.*, 2004 ▸).

## Synthesis and crystallization   

The reagents Cd[S_2_CN(^*i*^Pr)CH_2_CH_2_OH]_2_ (Tan *et al.*, 2013 ▸; 206 mg, 0.44 mmol) and piperazine (Sigma–Aldrich; 76 mg, 0.43 mmol) were dissolved in chloro­form (15 ml) and aceto­nitrile (5 ml), respectively. The latter solution was added dropwise into the chloro­form solution and the resulting mixture was stirred for 1 h at room temperature. Slow evaporation of the clear solution yielded colourless crystals. M.p. 415–417 K. IR (cm^−1^): ν (O—H) 3281, ν(C—N) 1442, ν (C—O) 1169, ν (C—S) 1029.

## Refinement   

Crystal data, data collection and structure refinement details are summarized in Table 5 ▸. The carbon-bound H atoms were placed in calculated positions (C—H = 0.98–1.00 Å) and were included in the refinement in the riding-model approximation, with *U*_iso_(H) set to 1.2*U*_eq_(C). The oxygen- and nitro­gen-bound H atoms were located in a difference Fourier map but were refined with a distance restraints of O—H = 0.84±0.01 Å and N—H = 0.88±0.01 Å, and with *U*_iso_(H) set to 1.5*U*_eq_(O) and 1.2*U*_eq_(N).

## Supplementary Material

Crystal structure: contains datablock(s) I, global. DOI: 10.1107/S2056989016000165/hb7558sup1.cif

Structure factors: contains datablock(s) I. DOI: 10.1107/S2056989016000165/hb7558Isup2.hkl

CCDC reference: 1445316

Additional supporting information:  crystallographic information; 3D view; checkCIF report

## Figures and Tables

**Table 1:** Selected geometric parameters (Å, °)

Cd—S1	2.5503 (6)	C1—S1	1.732 (2)
Cd—S2	2.6580 (8)	C1—S2	1.717 (2)
Cd—S3	2.5446 (6)	C7—S3	1.727 (2)
Cd—S4	2.7461 (8)	C7—S4	1.718 (2)
Cd—N3	2.3102 (17)		
			
S1—Cd—S2	69.63 (2)	S2—Cd—S4	156.230 (18)
S1—Cd—S3	145.16 (2)	S2—Cd—N3	104.12 (4)
S1—Cd—S4	101.38 (2)	S3—Cd—S4	68.29 (2)
S1—Cd—N3	116.43 (5)	S3—Cd—N3	98.30 (5)
S2—Cd—S3	105.98 (2)	S4—Cd—N3	99.57 (4)

**Table 2:** Hydrogen-bond geometry (Å, °)

*D*—H⋯*A*	*D*—H	H⋯*A*	*D*⋯*A*	*D*—H⋯*A*
O1—H1*O*⋯O2^i^	0.82 (2)	1.91 (2)	2.673 (2)	155 (2)
O2—H2*O*⋯N4^ii^	0.83 (2)	1.93 (2)	2.749 (2)	169 (3)
N3—H3*N*⋯O1^iii^	0.87 (2)	2.05 (2)	2.894 (2)	165 (2)
N4—H4*N*⋯O2^iv^	0.87 (1)	2.67 (1)	3.501 (3)	162 (1)
C4—H4⋯S3^v^	1.00	2.82	3.641 (3)	140
C10—H10⋯S1^vi^	1.00	2.83	3.659 (3)	141

Symmetry codes: (i) 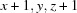; (ii) 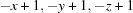; (iii) 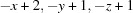; (iv) 
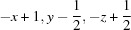
; (v) 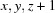; (vi) 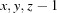.

**Table 3:** Percentage contribution of the different inter­molecular contacts to the Hirshfeld surface

Contact	Contribution
H⋯H	67.5
S⋯H/H⋯S	17.4
O⋯H/H⋯O	7.9
C⋯H/H⋯C	3.2
N⋯H/H⋯N	2.3
S⋯S	1.0
Cd⋯H/H⋯Cd	0.6
Others	0.1

**Table 4:** Enrichment ratios (ER)

Contact	ER
H⋯H	0.98
O⋯H	1.2
N⋯H	1.2
C⋯H	1.2
S⋯H	1.1
S⋯S	1.1

**Table 5:** Experimental details

Crystal data	
Chemical formula	[Cd(C_6_H_12_NOS_2_)_2_(C_4_H_10_N_2_)]
*M*_r_	555.11
Crystal system, space group	Monoclinic, *P*2_1_/*c*
Temperature (K)	100
*a*, *b*, *c* (Å)	15.341 (3), 16.9915 (7), 9.0308 (8)
β (°)	100.620 (16)
*V* (Å^3^)	2313.7 (5)
*Z*	4
Radiation type	Mo *K*α
μ (mm^−1^)	1.32
Crystal size (mm)	0.35 × 0.30 × 0.25

Data collection	
Diffractometer	Agilent SuperNova Dual diffractometer with Atlas detector
Absorption correction	Multi-scan (*CrysAlis PRO*; Agilent, 2011 ▸)
*T*_min_, *T*_max_	0.805, 1.000
No. of measured, independent and observed [*I* > 2σ(*I*)] reflections	19918, 5336, 4483
*R*_int_	0.035
(sin θ/λ)_max_ (Å^−1^)	0.651

Refinement	
*R*[*F* ^2^ > 2σ(*F* ^2^)], *wR*(*F* ^2^), *S*	0.026, 0.058, 1.03
No. of reflections	5336
No. of parameters	260
No. of restraints	4
Δρ_max_, Δρ_min_ (e Å^−3^)	0.45, −0.45

Computer programs: *CrysAlis PRO* (Agilent, 2011 ▸), *SHELXS97* (Sheldrick, 2008 ▸), *SHELXL2014*/7 (Sheldrick, 2015 ▸), *ORTEP-3 for Windows* (Farrugia, 2012 ▸), *DIAMOND* (Brandenburg, 2006 ▸) and *publCIF* (Westrip, 2010 ▸).

## supplementary crystallographic information

### Crystal data

**Table:**

[Cd(C_6_H_12_NOS_2_)_2_(C_4_H_10_N_2_)]	*F*(000) = 1144
*M**_r_* = 555.11	*D*_x_ = 1.594 Mg m^−^^3^
Monoclinic, *P*2_1_/*c*	Mo *K*α radiation, λ = 0.71073 Å
*a* = 15.341 (3) Å	Cell parameters from 7141 reflections
*b* = 16.9915 (7) Å	θ = 2.3–27.5°
*c* = 9.0308 (8) Å	µ = 1.32 mm^−^^1^
β = 100.620 (16)°	*T* = 100 K
*V* = 2313.7 (5) Å^3^	Block, colourless
*Z* = 4	0.35 × 0.30 × 0.25 mm

### Data collection

**Table:**

Agilent SuperNova Dual diffractometer with Atlas detector	5336 independent reflections
Radiation source: SuperNova (Mo) X-ray Source	4483 reflections with *I* > 2σ(*I*)
Mirror monochromator	*R*_int_ = 0.035
Detector resolution: 10.4041 pixels mm^-1^	θ_max_ = 27.6°, θ_min_ = 2.6°
ω scan	*h* = −19→19
Absorption correction: multi-scan (*CrysAlis PRO*; Agilent, 2011)	*k* = −22→21
*T*_min_ = 0.805, *T*_max_ = 1.000	*l* = −11→11
19918 measured reflections	

### Refinement

**Table:**

Refinement on *F*^2^	4 restraints
Least-squares matrix: full	Hydrogen site location: mixed
*R*[*F*^2^ > 2σ(*F*^2^)] = 0.026	*w* = 1/[σ^2^(*F*_o_^2^) + (0.0205*P*)^2^ + 0.8997*P*] where *P* = (*F*_o_^2^ + 2*F*_c_^2^)/3
*wR*(*F*^2^) = 0.058	(Δ/σ)_max_ = 0.001
*S* = 1.03	Δρ_max_ = 0.45 e Å^−^^3^
5336 reflections	Δρ_min_ = −0.45 e Å^−^^3^
260 parameters	

### Special details

**Table:**

Geometry. All esds (except the esd in the dihedral angle between two l.s. planes) are estimated using the full covariance matrix. The cell esds are taken into account individually in the estimation of esds in distances, angles and torsion angles; correlations between esds in cell parameters are only used when they are defined by crystal symmetry. An approximate (isotropic) treatment of cell esds is used for estimating esds involving l.s. planes.

### Fractional atomic coordinates and isotropic or equivalent isotropic displacement parameters (Å^2^)

**Table:**

	*x*	*y*	*z*	*U*_iso_*/*U*_eq_	
Cd	0.76764 (2)	0.52627 (2)	0.47410 (2)	0.01437 (5)	
S1	0.78258 (4)	0.56986 (3)	0.74785 (6)	0.01975 (12)	
S2	0.93633 (3)	0.55625 (3)	0.59115 (6)	0.01631 (12)	
S3	0.74500 (4)	0.57217 (3)	0.20148 (6)	0.02150 (13)	
S4	0.59316 (3)	0.56165 (3)	0.36324 (6)	0.01639 (12)	
O1	1.18537 (10)	0.63462 (9)	0.85852 (17)	0.0188 (3)	
H1O	1.2282 (12)	0.6228 (14)	0.923 (2)	0.028*	
O2	0.33805 (10)	0.64272 (9)	0.05591 (18)	0.0198 (3)	
H2O	0.3353 (17)	0.6586 (14)	0.1420 (15)	0.030*	
N1	0.94525 (11)	0.62212 (10)	0.86113 (19)	0.0144 (4)	
N2	0.58291 (11)	0.62440 (10)	0.0898 (2)	0.0146 (4)	
N3	0.76459 (11)	0.39219 (10)	0.4310 (2)	0.0145 (4)	
H3N	0.7843 (14)	0.3924 (13)	0.3467 (16)	0.017*	
N4	0.69255 (12)	0.31320 (10)	0.6653 (2)	0.0178 (4)	
H4N	0.6902 (15)	0.2653 (7)	0.631 (2)	0.021*	
C1	0.89375 (14)	0.58600 (11)	0.7450 (2)	0.0139 (4)	
C2	1.03323 (13)	0.65184 (12)	0.8466 (2)	0.0161 (4)	
H2A	1.0464	0.6989	0.9115	0.019*	
H2B	1.0309	0.6689	0.7411	0.019*	
C3	1.10896 (13)	0.59392 (12)	0.8877 (3)	0.0177 (5)	
H3A	1.1165	0.5789	0.9952	0.021*	
H3B	1.0980	0.5458	0.8252	0.021*	
C4	0.90981 (14)	0.64845 (13)	0.9957 (2)	0.0183 (5)	
H4	0.8542	0.6181	0.9977	0.022*	
C5	0.88517 (16)	0.73502 (14)	0.9818 (3)	0.0277 (6)	
H5A	0.8426	0.7437	0.8881	0.042*	
H5B	0.9386	0.7665	0.9806	0.042*	
H5C	0.8584	0.7507	1.0678	0.042*	
C6	0.97444 (15)	0.63025 (14)	1.1409 (3)	0.0255 (5)	
H6A	0.9920	0.5748	1.1413	0.038*	
H6B	0.9458	0.6407	1.2274	0.038*	
H6C	1.0271	0.6636	1.1474	0.038*	
C7	0.63461 (14)	0.58907 (11)	0.2067 (2)	0.0148 (4)	
C8	0.49551 (13)	0.65624 (12)	0.1036 (2)	0.0156 (4)	
H8A	0.4834	0.7030	0.0377	0.019*	
H8B	0.4981	0.6741	0.2087	0.019*	
C9	0.41828 (14)	0.59923 (12)	0.0634 (3)	0.0183 (5)	
H9A	0.4198	0.5741	−0.0350	0.022*	
H9B	0.4222	0.5575	0.1408	0.022*	
C10	0.61846 (14)	0.64931 (12)	−0.0452 (2)	0.0173 (5)	
H10	0.6722	0.6166	−0.0493	0.021*	
C11	0.64800 (16)	0.73483 (13)	−0.0293 (3)	0.0253 (5)	
H11A	0.6902	0.7417	0.0654	0.038*	
H11B	0.5963	0.7687	−0.0292	0.038*	
H11C	0.6766	0.7492	−0.1140	0.038*	
C12	0.55260 (15)	0.63515 (13)	−0.1906 (3)	0.0230 (5)	
H12A	0.5319	0.5805	−0.1936	0.034*	
H12B	0.5815	0.6451	−0.2769	0.034*	
H12C	0.5019	0.6707	−0.1950	0.034*	
C13	0.67256 (14)	0.36241 (12)	0.4019 (2)	0.0163 (4)	
H13A	0.6711	0.3086	0.3594	0.020*	
H13B	0.6349	0.3967	0.3277	0.020*	
C14	0.63728 (14)	0.36126 (12)	0.5478 (2)	0.0173 (5)	
H14A	0.6347	0.4158	0.5851	0.021*	
H14B	0.5761	0.3400	0.5281	0.021*	
C15	0.78608 (14)	0.33777 (13)	0.6863 (2)	0.0187 (5)	
H15A	0.8225	0.3006	0.7562	0.022*	
H15B	0.7921	0.3905	0.7338	0.022*	
C16	0.82184 (14)	0.34096 (12)	0.5409 (2)	0.0180 (5)	
H16A	0.8831	0.3619	0.5610	0.022*	
H16B	0.8234	0.2873	0.4988	0.022*	

### Atomic displacement parameters (Å^2^)

**Table:**

	*U*^11^	*U*^22^	*U*^33^	*U*^12^	*U*^13^	*U*^23^
Cd	0.01554 (9)	0.01362 (8)	0.01268 (9)	−0.00077 (6)	−0.00071 (6)	0.00017 (6)
S1	0.0124 (3)	0.0291 (3)	0.0181 (3)	−0.0043 (2)	0.0038 (2)	−0.0087 (2)
S2	0.0142 (3)	0.0209 (3)	0.0139 (3)	0.0001 (2)	0.0027 (2)	−0.0039 (2)
S3	0.0130 (3)	0.0336 (3)	0.0182 (3)	0.0052 (2)	0.0036 (2)	0.0104 (2)
S4	0.0155 (3)	0.0184 (3)	0.0156 (3)	0.0017 (2)	0.0038 (2)	0.0025 (2)
O1	0.0119 (8)	0.0277 (8)	0.0162 (8)	−0.0023 (6)	0.0008 (6)	0.0011 (7)
O2	0.0132 (8)	0.0279 (9)	0.0177 (8)	0.0031 (6)	0.0011 (7)	−0.0014 (7)
N1	0.0130 (9)	0.0167 (9)	0.0142 (9)	−0.0016 (7)	0.0042 (7)	−0.0024 (7)
N2	0.0121 (9)	0.0155 (9)	0.0159 (9)	0.0026 (7)	0.0014 (7)	0.0016 (7)
N3	0.0145 (9)	0.0171 (9)	0.0123 (9)	0.0004 (7)	0.0035 (7)	0.0018 (7)
N4	0.0212 (10)	0.0138 (9)	0.0186 (10)	−0.0017 (7)	0.0045 (8)	0.0013 (7)
C1	0.0150 (11)	0.0109 (10)	0.0152 (11)	0.0019 (8)	0.0015 (8)	0.0023 (8)
C2	0.0135 (11)	0.0188 (11)	0.0161 (11)	−0.0049 (8)	0.0026 (9)	−0.0011 (9)
C3	0.0134 (11)	0.0214 (11)	0.0175 (11)	−0.0026 (8)	0.0010 (9)	0.0003 (9)
C4	0.0154 (11)	0.0246 (12)	0.0157 (11)	−0.0044 (9)	0.0050 (9)	−0.0069 (9)
C5	0.0265 (13)	0.0304 (13)	0.0271 (14)	0.0040 (10)	0.0070 (11)	−0.0108 (11)
C6	0.0230 (13)	0.0384 (14)	0.0157 (12)	−0.0078 (10)	0.0050 (10)	−0.0040 (10)
C7	0.0146 (11)	0.0122 (10)	0.0170 (11)	−0.0007 (8)	0.0014 (9)	−0.0008 (8)
C8	0.0153 (11)	0.0138 (10)	0.0176 (11)	0.0026 (8)	0.0027 (9)	0.0011 (8)
C9	0.0151 (11)	0.0205 (11)	0.0192 (12)	0.0026 (8)	0.0027 (9)	0.0003 (9)
C10	0.0145 (11)	0.0220 (11)	0.0153 (11)	0.0028 (8)	0.0026 (9)	0.0050 (9)
C11	0.0255 (13)	0.0265 (13)	0.0231 (13)	−0.0060 (10)	0.0025 (10)	0.0072 (10)
C12	0.0210 (12)	0.0279 (12)	0.0184 (12)	0.0024 (9)	−0.0004 (10)	0.0031 (10)
C13	0.0167 (11)	0.0151 (10)	0.0160 (11)	−0.0013 (8)	0.0000 (9)	−0.0019 (8)
C14	0.0145 (11)	0.0159 (10)	0.0209 (12)	−0.0009 (8)	0.0016 (9)	0.0000 (9)
C15	0.0181 (11)	0.0197 (11)	0.0173 (12)	0.0040 (9)	0.0008 (9)	0.0052 (9)
C16	0.0157 (11)	0.0175 (11)	0.0203 (12)	0.0031 (8)	0.0023 (9)	0.0020 (9)

### Geometric parameters (Å, º)

**Table:**

Cd—S1	2.5503 (6)	C4—H4	1.0000
Cd—S2	2.6580 (8)	C5—H5A	0.9800
Cd—S3	2.5446 (6)	C5—H5B	0.9800
Cd—S4	2.7461 (8)	C5—H5C	0.9800
Cd—N3	2.3102 (17)	C6—H6A	0.9800
C1—S1	1.732 (2)	C6—H6B	0.9800
C1—S2	1.717 (2)	C6—H6C	0.9800
C7—S3	1.727 (2)	C8—C9	1.522 (3)
C7—S4	1.718 (2)	C8—H8A	0.9900
O1—C3	1.427 (2)	C8—H8B	0.9900
O1—H1O	0.821 (10)	C9—H9A	0.9900
O2—C9	1.426 (2)	C9—H9B	0.9900
O2—H2O	0.831 (10)	C10—C12	1.521 (3)
N1—C1	1.340 (3)	C10—C11	1.521 (3)
N1—C2	1.470 (3)	C10—H10	1.0000
N1—C4	1.488 (3)	C11—H11A	0.9800
N2—C7	1.340 (3)	C11—H11B	0.9800
N2—C8	1.472 (3)	C11—H11C	0.9800
N2—C10	1.487 (3)	C12—H12A	0.9800
N3—C13	1.477 (3)	C12—H12B	0.9800
N3—C16	1.481 (3)	C12—H12C	0.9800
N3—H3N	0.869 (9)	C13—C14	1.514 (3)
N4—C15	1.473 (3)	C13—H13A	0.9900
N4—C14	1.477 (3)	C13—H13B	0.9900
N4—H4N	0.868 (9)	C14—H14A	0.9900
C2—C3	1.516 (3)	C14—H14B	0.9900
C2—H2A	0.9900	C15—C16	1.515 (3)
C2—H2B	0.9900	C15—H15A	0.9900
C3—H3A	0.9900	C15—H15B	0.9900
C3—H3B	0.9900	C16—H16A	0.9900
C4—C5	1.518 (3)	C16—H16B	0.9900
C4—C6	1.523 (3)		
			
S1—Cd—S2	69.63 (2)	C4—C6—H6C	109.5
S1—Cd—S3	145.16 (2)	H6A—C6—H6C	109.5
S1—Cd—S4	101.38 (2)	H6B—C6—H6C	109.5
S1—Cd—N3	116.43 (5)	N2—C7—S4	120.98 (16)
S2—Cd—S3	105.98 (2)	N2—C7—S3	119.60 (16)
S2—Cd—S4	156.230 (18)	S4—C7—S3	119.42 (12)
S2—Cd—N3	104.12 (4)	N2—C8—C9	115.29 (17)
S3—Cd—S4	68.29 (2)	N2—C8—H8A	108.5
S3—Cd—N3	98.30 (5)	C9—C8—H8A	108.5
S4—Cd—N3	99.57 (4)	N2—C8—H8B	108.5
C1—S1—Cd	86.81 (7)	C9—C8—H8B	108.5
C1—S2—Cd	83.72 (7)	H8A—C8—H8B	107.5
C7—S3—Cd	89.09 (8)	O2—C9—C8	107.97 (17)
C7—S4—Cd	82.85 (7)	O2—C9—H9A	110.1
C3—O1—H1O	109.0 (18)	C8—C9—H9A	110.1
C9—O2—H2O	108.4 (18)	O2—C9—H9B	110.1
C1—N1—C2	120.42 (18)	C8—C9—H9B	110.1
C1—N1—C4	121.68 (17)	H9A—C9—H9B	108.4
C2—N1—C4	116.70 (16)	N2—C10—C12	112.18 (18)
C7—N2—C8	120.95 (18)	N2—C10—C11	110.04 (18)
C7—N2—C10	121.38 (17)	C12—C10—C11	111.81 (18)
C8—N2—C10	116.24 (16)	N2—C10—H10	107.5
C13—N3—C16	110.33 (16)	C12—C10—H10	107.5
C13—N3—Cd	110.91 (12)	C11—C10—H10	107.5
C16—N3—Cd	118.40 (13)	C10—C11—H11A	109.5
C13—N3—H3N	108.7 (15)	C10—C11—H11B	109.5
C16—N3—H3N	109.3 (15)	H11A—C11—H11B	109.5
Cd—N3—H3N	98.3 (15)	C10—C11—H11C	109.5
C15—N4—C14	110.64 (16)	H11A—C11—H11C	109.5
C15—N4—H4N	106.7 (16)	H11B—C11—H11C	109.5
C14—N4—H4N	106.4 (15)	C10—C12—H12A	109.5
N1—C1—S2	120.73 (16)	C10—C12—H12B	109.5
N1—C1—S1	120.06 (16)	H12A—C12—H12B	109.5
S2—C1—S1	119.20 (12)	C10—C12—H12C	109.5
N1—C2—C3	115.48 (17)	H12A—C12—H12C	109.5
N1—C2—H2A	108.4	H12B—C12—H12C	109.5
C3—C2—H2A	108.4	N3—C13—C14	109.43 (17)
N1—C2—H2B	108.4	N3—C13—H13A	109.8
C3—C2—H2B	108.4	C14—C13—H13A	109.8
H2A—C2—H2B	107.5	N3—C13—H13B	109.8
O1—C3—C2	105.00 (16)	C14—C13—H13B	109.8
O1—C3—H3A	110.7	H13A—C13—H13B	108.2
C2—C3—H3A	110.7	N4—C14—C13	112.50 (18)
O1—C3—H3B	110.7	N4—C14—H14A	109.1
C2—C3—H3B	110.7	C13—C14—H14A	109.1
H3A—C3—H3B	108.8	N4—C14—H14B	109.1
N1—C4—C5	110.29 (18)	C13—C14—H14B	109.1
N1—C4—C6	111.36 (17)	H14A—C14—H14B	107.8
C5—C4—C6	112.35 (18)	N4—C15—C16	113.49 (18)
N1—C4—H4	107.5	N4—C15—H15A	108.9
C5—C4—H4	107.5	C16—C15—H15A	108.9
C6—C4—H4	107.5	N4—C15—H15B	108.9
C4—C5—H5A	109.5	C16—C15—H15B	108.9
C4—C5—H5B	109.5	H15A—C15—H15B	107.7
H5A—C5—H5B	109.5	N3—C16—C15	109.71 (17)
C4—C5—H5C	109.5	N3—C16—H16A	109.7
H5A—C5—H5C	109.5	C15—C16—H16A	109.7
H5B—C5—H5C	109.5	N3—C16—H16B	109.7
C4—C6—H6A	109.5	C15—C16—H16B	109.7
C4—C6—H6B	109.5	H16A—C16—H16B	108.2
H6A—C6—H6B	109.5		
			
C2—N1—C1—S2	12.9 (2)	Cd—S4—C7—N2	−173.40 (17)
C4—N1—C1—S2	179.99 (15)	Cd—S4—C7—S3	5.59 (11)
C2—N1—C1—S1	−166.32 (14)	Cd—S3—C7—N2	173.01 (16)
C4—N1—C1—S1	0.8 (3)	Cd—S3—C7—S4	−5.99 (11)
Cd—S2—C1—N1	−171.64 (16)	C7—N2—C8—C9	−90.4 (2)
Cd—S2—C1—S1	7.59 (11)	C10—N2—C8—C9	103.1 (2)
Cd—S1—C1—N1	171.36 (16)	N2—C8—C9—O2	−169.59 (17)
Cd—S1—C1—S2	−7.88 (11)	C7—N2—C10—C12	141.10 (19)
C1—N1—C2—C3	−89.4 (2)	C8—N2—C10—C12	−52.4 (2)
C4—N1—C2—C3	102.9 (2)	C7—N2—C10—C11	−93.8 (2)
N1—C2—C3—O1	177.54 (16)	C8—N2—C10—C11	72.7 (2)
C1—N1—C4—C5	−96.9 (2)	C16—N3—C13—C14	59.9 (2)
C2—N1—C4—C5	70.7 (2)	Cd—N3—C13—C14	−73.30 (17)
C1—N1—C4—C6	137.7 (2)	C15—N4—C14—C13	52.8 (2)
C2—N1—C4—C6	−54.8 (2)	N3—C13—C14—N4	−57.4 (2)
C8—N2—C7—S4	13.3 (3)	C14—N4—C15—C16	−51.7 (2)
C10—N2—C7—S4	179.18 (14)	C13—N3—C16—C15	−58.4 (2)
C8—N2—C7—S3	−165.65 (14)	Cd—N3—C16—C15	70.83 (19)
C10—N2—C7—S3	0.2 (3)	N4—C15—C16—N3	54.8 (2)

### Hydrogen-bond geometry (Å, º)

**Table:**

*D*—H···*A*	*D*—H	H···*A*	*D*···*A*	*D*—H···*A*
O1—H1*O*···O2^i^	0.82 (2)	1.91 (2)	2.673 (2)	155 (2)
O2—H2*O*···N4^ii^	0.83 (2)	1.93 (2)	2.749 (2)	169 (3)
N3—H3*N*···O1^iii^	0.87 (2)	2.05 (2)	2.894 (2)	165 (2)
N4—H4*N*···O2^iv^	0.87 (1)	2.67 (1)	3.501 (3)	162 (1)
C4—H4···S3^v^	1.00	2.82	3.641 (3)	140
C10—H10···S1^vi^	1.00	2.83	3.659 (3)	141

Symmetry codes: (i) *x*+1, *y*, *z*+1; (ii) −*x*+1, −*y*+1, −*z*+1; (iii) −*x*+2, −*y*+1, −*z*+1; (iv) −*x*+1, *y*−1/2, −*z*+1/2; (v) *x*, *y*, *z*+1; (vi) *x*, *y*, *z*−1.
